# Cervical Cancer Screening: Assessment of Perception and Utilization of Services among Health Workers in Low Resource Setting

**DOI:** 10.1155/2019/6505482

**Published:** 2019-02-03

**Authors:** C. C. Ifemelumma, C. C. Anikwe, B. C. Okorochukwu, F. A. Onu, J. A. Obuna, B. N. Ejikeme, O. P. Ezeonu

**Affiliations:** ^1^Department of Obstetrics and Gynaecology, Federal Teaching Hospital Abakaliki Ebonyi State, PMB 102 Abakaliki, Ebonyi State, Nigeria; ^2^Department of Obstetrics and Gynaecology, Federal Medical Centre Owerri, P. O. Box 1010, Owerri, Imo State, Nigeria

## Abstract

**Background:**

Cervical cancer is a preventable disease and the most common cancer among women in Nigeria.

**Objectives:**

To appraise the perception and utilization of cervical cancer screening services among female nurses in Federal Teaching hospital, Abakaliki.

**Materials and Methods:**

A cross-sectional study was done using semi-structured questionnaires on 408 consenting female nurses. Data was represented using simple percentages, charts, and Chi-square.

**Results:**

Of the 408 questionnaires shared, 388 were correctly and completely filled and analyzed. The respondents in this study showed good knowledge of cervical cancer as all (388) were aware that cervical cancer is a preventable disease of public health concern. Majority of them, 179 (46.1%) were between 21 and 30 years of age. The most common symptom of cervical cancer identified was postcoital bleeding (57%). Nursing training was the most common (73.5%) source of cervical cancer information. Utilization of cervical cancer screening was poor in this study as only 20.6% of the respondents had ever undergone screening. The most common reason for nonscreening was that they have not thought of it (28.4%).

**Conclusion:**

Despite the high level of awareness of cervical cancer screening, utilization remains low. There is, therefore, the need for cervical cancer education for the nurses to help improve utilization.

## 1. Introduction

Cancer of the cervix, a potentially preventable disease [1], is the second most common cancer among women worldwide [2, 3] and the most common malignancy of the female genital tract in the developing countries [4, 5]. Worldwide, cervical cancer accounts for about 500,000 new diagnoses and 273,000 deaths every year, of the new cases 80% occur in the developing countries [2]. About 85% of the 250,000 deaths recorded yearly from cervical cancer occur in developing countries like Nigeria where the majority of cases present in the late stages of the disease [6, 7]. In Nigeria, the national incidence of cancer of the cervix is 250/100,000 [7]. Each year approximately, 10,000 women develop cervical cancer, and about 8,000 women die from cervical cancer in Nigeria [8]. It is worrisome as all sexually active women are at risk for the development of cervical cancer.

Sexually transmitted Human Papilloma Virus (HPV) infection leads to the development of cervical intraepithelial neoplasia and cervical cancer. Women with many sexual partners and those whose partners have had many sexual consorts or have been previously exposed to the virus are most at risk of developing the disease [7]. Early detection and prompt treatment of precancerous conditions provide the best possible protection against cancer [9]. In developed countries with well-established screening programs, the incidences of cervical cancer have been reduced by 70-90%. Sporadic screening is being carried out in Nigeria using opportunist method for those who visit certain clinics. Also, there is no standard policy or protocol for cervical cancer screening in Nigeria [10]. The different methods of cervical cancer screening include Papanicolaou (Pap) smear, visual inspection of the cervix with acetic acid (VIA), HPV DNA test, and colposcopy [11]. Colposcopy is not used as a primary screening test but it is combined with other tests.

The high burden of cervical cancer in developing countries, like Nigeria, is due both to a high prevalence of HPV infection and the lack of effective cervical cancer screening programmes [10]. In cases where effective screening programmes are available, poor knowledge and negative health-seeking behavior of the populace have led to poor utilization of such services [10]. An important strategy towards the reduction of the incidence and mortality associated with cervical cancer is by increasing the screening rate of women that have not screened or those that screen infrequently [12]. Knowledge about cancer of the cervix and its screening is important in screening uptake. Women with low levels of knowledge about cervical cancer and its prevention are less likely to access screening services [3, 7, 9, 13]. Previous studies done among female health workers have shown good knowledge of cervical cancer; however, cervical cancer screening attendance rates are still far from satisfactory in most countries [5, 14, 15]. Nurses play a major role in promoting health care services and in enlightening the public on many health-related issues and their knowledge and attitude on health-related issues are crucial in gaining and promoting patients' uptake of care. They also help to improve women's confidence. This study was embarked upon to fill a knowledge gap since no such has been done in our centre. Our aim is to appraise the perception and utilization of cervical cancer screening services among female nurses in a Federal Teaching Hospital Abakaliki. We specifically assessed the knowledge of the nurses towards cervical cancer, the percentage of female nurses utilizing cervical cancer screening services, and the factors influencing utilization of cervical cancer screening services.

## 2. Materials and Methods

### 2.1. Study Area

The study was carried out among female nurses of the Federal Teaching Hospital, Abakaliki. The hospital is a referral tertiary centre and the only teaching hospital in the state. It was formed in 2012 from the merger of the former Ebonyi State Teaching Hospital and the then Federal Medical Centre, Abakaliki. The hospital has an active Well Woman Centre which offers screening for genital tract cancer as one of its activities. There are 1306 female nurses working in Federal Teaching Hospital, Abakaliki (FETHA), in all departments.

### 2.2. Study Design

This was a descriptive cross-sectional study.

### 2.3. Sample Size

The sample size (N_0_) was calculated using

(1) $$\begin{array}{c} N_{o}=\frac{Z^{2}pq}{e^{2}} \end{array}$$

where Z^2^ is a constant = 1.96.

e: the desired level of precision also known as sampling error: 5%.

p: prevalence of utilization of cervical cancer screening services from similar study^9^ = 0.35.

q: 1-p.

(2) $$\begin{array}{c} N_{o}=\frac{{\left(1.96\right)}^{2}\times 0.35\times 0.65}{{\left(0.05\right)}^{2}}=349.58 \end{array}$$

Attrition rate of 20% was added to the sample size, i.e., 350 + 70 = 420. Therefore the sample size of 420 was used.

### 2.4. Sample Technique and Data Collection

Data were collected using self-administered semistructured questionnaire. The questionnaire had 3 parts; the first part assessed the sociodemographic characteristics of the respondents while the other 2 parts assessed their perception and utilization of cervical cancer screening, respectively. Proportionate and systematic sampling techniques were employed. Six (6) clinical departments, namely, Obstetrics and Gynecology, Family Medicine, Internal Medicine, General Surgery, Ophthalmology, and Paediatrics, were selected by balloting from the total number of the 13 clinical departments in the facility. A proportionate allocation of the 420 questionnaires to the female nurses in the respective departments was done based on the total number of female nurses in each of the selected departments; i.e., 1 questionnaire was allotted for every 2 nurses in each of the respective department. This resulted in 112, 49, 60, 78, 41, and 80 questionnaires allotted to Obstetrics and Gynecology, Family Medicine, Internal Medicine, General Surgery, Ophthalmology, and Paediatrics Departments, respectively. The allotted questionnaires were then shared randomly, using a systematic sampling method using the nurses on the departmental duty register as the sampling frame. Care was taken to avoid readministering the questionnaire to those that had previously been administered. This was done by simply asking if they had previously answered to this particular questionnaire. The purpose of the study was explained and consent obtained. Five (5) medical students were trained to administer the questionnaires. The questionnaires were retrieved from each respondent immediately after completion and they were reviewed for completeness. The male nurses were exempted.

### 2.5. Data Analysis

The data obtained were analyzed using IBM SPSS Statistics version 20 (IBM Corp., Armonk, NY, USA). The data obtained were reclassified for easy analysis, age-grouped into ≤ 40 years and > 40 years, marital status into married and unmarried, parity into ≤ 4 and > 4, and duration of service into < 5 years and ≥ 5 years. The results were presented in frequency tables, charts, and Chi-square contingency tables.

### 2.6. Ethical Consideration

Permission to carry out this research was sought and obtained from the Research and Ethics Committee of the Federal Teaching Hospital Abakaliki. The ethical approval number is FETHA/REC VOL l /2014/189.

## 3. Results

In this study, 388 out of 420 questionnaires were correctly completed and returned which gave a retrieval rate of 92.4%. As in Table 1, the majority of the respondents, 179 (46.1%), were between 21 and 30 years of age and the fewest, 8 (2.1%), were between ages of 51 to 60 years. Of the respondents, 209 (53.9%) were married, while 160 (41.2%) were single. Most of (190, 46.1%) the respondents belong to Para 1-4. Nurses with less than 5 years of working experience made up the largest proportion (47.2%).

From Table 2, the respondents in this study showed good knowledge of cervical cancer as all were aware that cervical cancer is a preventable disease of public health concern. The most common symptom of cancer of the cervix identified was posted coital bleeding (57.7%). The majority (72.4%) of the respondents knew that history of Human Papilloma Virus (HPV) infection is a risk factor although only 86.2% (335) correctly identified HPV as the primary cause of cervical cancer (Figure 2). Less than 10% identified poor hygiene and alcohol intake, respectively, as risk factors for cancer of the cervix. Most of the respondents (89.2%) identified Pap smear as a screening modality, while only 74.5% of the respondents were aware of the HPV vaccine. Two hundred and thirty-two (59.8%) of the respondents perceived being above 21 years of age or sexually active for the last 3 years (whichever is earlier) as an indication to be screened for cancer cervix.

**Figure 1:**
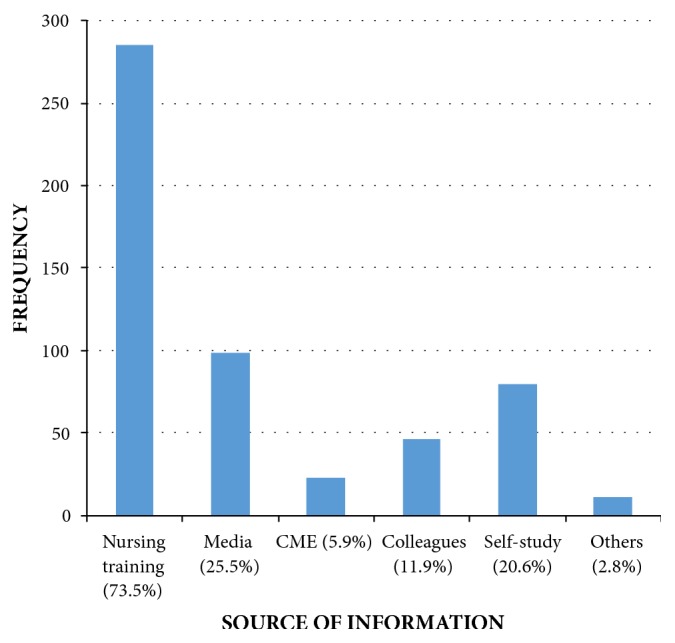
Nurses' sources of cervical cancer information. CME: continuing medical education.

**Figure 2:**
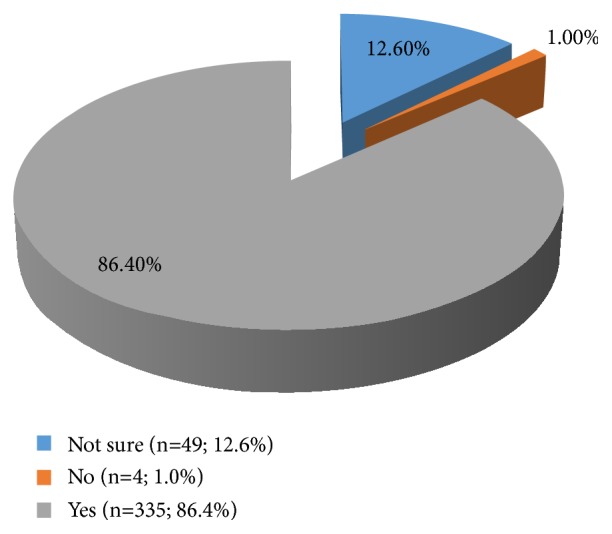
HPV as primary cause of cancer of the cervix.

In Figure 1, nursing training is the commonest source of information about cervical cancer.

**Figure 3:**
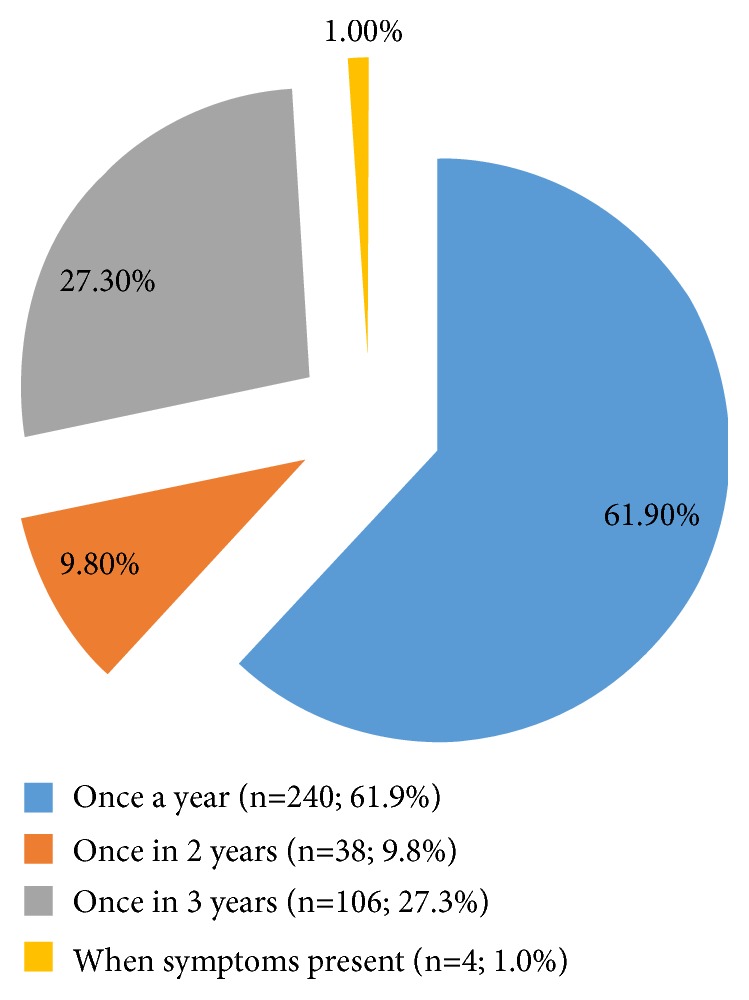
Frequency of cervical cancer screening.

Figure 3 shows the study population knowledge of cervical cancer screening frequency. It depicts poor knowledge as only 27.3% (106) of the respondents are aware that the screening frequency is once in 3 years.

Table 3 shows that only 20.6% of the respondents had ever undergone screening for cervical cancer. The most common reason for nonscreening was that they had not thought of it (28.4%) and 10.8% ascribe their nonscreening to the fear of the result. Among the 80 (20.6%) respondents that had ever been screened, 68 (85%) of them had been screened once while 12 (15%) had been screened twice.

Table 4 shows that majority 327 (84.3%) of the respondents were aware of a cervical cancer screening centre. Of these respondents, 168 (43.3%) do not routinely recommend cervical screening to others; majority 76.8% of them had no reason for not recommending the screening while 7.1% considered the procedure being painful as their reason

The result in Table 5 showed a significant association between age, marital status, duration of practice, and parity with the utilization of cervical cancer screening services by the respondents.

A cross-tabulation done using duration of practice, respondent age, marital status, and parity as dependent variables and having being screened of cervical cancer as independent variable shows that being 40 years or less (OR 1.18 95% CI 1.12–1.23), parity (OR 1.07 95% CI 1.04–1.10), married (OR 2.08 95% CI 1.85–2.34), and duration of practice of less than five (5) years (OR 2.99 95% CI 2.55-3.50) is associated with increased chances of being screened for cervical cancer among our respondents.

## 4. Discussion

Cervical cancer is largely a preventable disease [1, 16]. An important strategy towards the reduction of its burden in a developing country is by early diagnosis and management of the premalignant lesions of the disease; this would be achieved via screening of women at risk. The current study evaluated the perception and utilization of cervical cancer screening services among female nurses in Federal Teaching Hospital, Abakaliki. The respondents in our study had a good knowledge of cervical cancer, as all of them correctly identified cervical cancer as a preventable disease and the majority of them noted HPV as the primary cause. The high knowledge of cervical cancer found in this study is not surprising considering the profession of the respondents, as they are expected to be more knowledgeable than other women in the community. This is consistent with the previous studies done on this subject [4, 9]. Most of the respondent in our study acquired their knowledge about the disease from their training although mass media, self-study, and colleagues constituted other common sources of information. This is similar to the study done in Tanzania [17] and Lagos, Nigeria [4]. The most common symptom of cervical cancer identified in this study was postcoital vaginal bleeding (57.7%). Barely half of the respondents identified offensive vaginal discharge as a symptom of cervical cancer, while less than half knew that pelvic pain and irregular vaginal bleeding could be caused by cervical cancer. This finding is in tandem with the work of Urasa et al. [17] in Tanzania and which might be attributed to the nature of training given to our nurses which does not go in-depth in discussing the pathology of diseases.

Many nurses in this study had inadequate knowledge of risk factors for cervical cancer, as only 13.7% and 10.8% knew that smoking and impaired immunity, respectively, are risk factors. Low knowledge of association of smoking with cervical cancer is not surprising as smoking is not a common practice among Nigerian women but poor knowledge of the link between impaired immunity and cervical cancer is not encouraging in view of the evidence linking cervical cancer with HIV. Some of the respondents even had the erroneous belief that IUCD, poor hygiene, and alcohol could result in cervical cancer. This finding is similar to the findings in studies done in Tanzania [17, 18] and Lagos, Nigeria [4].

Development of any national cervical cancer prevention and control programme is pivotal in reducing morbidity and mortality associated with cervical cancer in sub-Saharan Africa [16] but this is still a distant dream in Nigeria [5]. It was therefore not surprising that only about one-quarter of the respondents knew the cervical cancer screening interval. The commonest known screening modality in this study was Pap smear (89.2%). Less than half of the respondents recognized VIA, cervical biopsy, HPV DNA testing, and colposcopy (13.7%) as screening modalities for cervical cancer. This finding is similar to the study done in Lagos, Nigeria [4], where colposcopy was the least recognized form of screening for cervical cancer. This low knowledge of other methods calls for more awareness campaign on screening modalities for cervical cancer so as to give people more knowledge of the disease.

The utilization of cervical cancer screening services in this study was poor; only 20.6% had undergone screening. The commonest reason given for this was that they had never thought of it. Other reasons given included not being sexually active, no time, and the fear of the result. This study further revealed that there is a significant association between some sociodemographic factors like the age of respondents, marital status, parity, and duration of practice with the utilization of cervical cancer screening services. This is similar to the finding by Awodele [4]. It might be explained by the fact that nearly half of the respondents were less than 30 years and had practiced for 5 years or less and therefore being young may perceive themselves as not susceptible to the development of precancerous/cancerous lesion and are therefore not bothered about such issues and hence not likely to use the service. These findings were slightly different from the study in Nnewi, Nigeria [5], where majority gave no reason for not testing; and of those that had reasons, the commonest were the fear of the result and not being susceptible to cervical cancer. These reasons need to be addressed in an intervention programme targeting this category of health workers, considering their number and the influence they have in the community. Institutional-based workshop and training on cervical cancer can be of enormous help in improving utilization of cervical cancer screening.

In conclusion, it is evident from this study that despite the high level of awareness of cervical cancer screening, utilization among nurses remains low. We, therefore, recommend that there is a need for the nurses to be actively involved in the cancer screening units and possibly undergo rotation through the unit to help douse the fear and uncertainties associated with the cervical cancer screening process. Cervical cancer screening education programmes need to be carried out among health care professionals at all levels, especially among nurses. The continuing education based programme provides an opportunity for doing this, especially as nurses constitute one of the most authoritative sources of information on health matters for the general populace, especially for women. As they become informed, they should be motivated to practice what they teach and lead by example. The adoption of cervical cancer screening as a preemployment test may also be considered.

## Figures and Tables

**Table 1:** Sociodemographic characteristics of study population.

VARIABLE	FREQUENCY	PERCENTAGE
Age (years)		
21-30	179	46.1
31-40	163	42
41-50	38	9.8
51-60	8	2.1
≥60	-	-
Total	388	100
Marital status		
Married	209	53.9
Single	106	41.2
Divorced	-	-
Widowed	15	3.9
Separated	4	1
Total	388	100
Ethnicity		
Igbo	350	90.2
Yoruba	15	3.9
Hausa	4	1
Others	19	4.9
Total	388	100
Religion		
Christianity	384	99
Islam	4	1
Traditional	-	-
Total	388	100
Parity		
Nullipara	179	46.1
1-4	190	49
≥5	19	4.9
Total	388	100
Duration of practice (years)		
<5	183	47.2
5-10	152	39.2
>10	53	13.6
Total	388	100

**Table 2:** Knowledge of cervical cancer and screening.

VARIABLES	FREQUENCY	PERCENTAGE
Symptoms		
No symptoms	99	25.5
Foul Offensive vaginal discharge	190	49
Irregular vaginal bleeding	167	43
Postcoital vaginal bleeding	224	57.7
Fever	38	9.8
Pelvic pain	106	27.3
Weight loss	76	19.6
Risk factors		
Sexual intercourse < 16 years	209	53.9
Multiple sexual partners	277	71.4
Smoking	53	13.7
History of HPV infection	281	72.4
Use of IUCD	34	8.8
Impaired immunity	42	10.8
Poor hygiene	30	7.7
Alcohol use	23	5.9
Screening modalities		
Pap smear	346	89.2
VIA	160	41.2
Cervical biopsy	179	46.1
HPVDNA test	99	25.5
Colposcopy	53	13.7
Who should be screened		
Above 21 years/sexually active	232	59.8
Married women only	4	1
Women above 30 years	152	39.2
If cervical changes are found early are they curable		
Yes	288	74.2
No	27	6.9
Not aware	23	5.9

Multiple answers allowed.

**Table 3:** Utilization of cervical cancer screening.

	FREQUENCY	PERCENTAGE
SCREENING		
Yes	80	20.6
No	308	79.4
REASON FOR NOT SCREENING		
No time	50	12.9
Fear of result	42	10.8
Procedure being cumbersome	15	3.9
Not sexually active	50	12.9
Cost	0	0
Not thought of it	110	28.4
Lack of awareness of test	11	2.8
Don't know where a test is done	15	3.9
Others	30	7.7

**Table 4:** Nurses' attitude towards recommendation for cervical cancer screening.

	Frequency	Percentage
Do not routinely recommend		

Screening		
No	168	43.3
Yes	220	56.7

Reasons for not recommending screening		

Procedure is painful	12	7.1
Females 21 years old are safe	8	4.8
The test could be dangerous and Risky	4	2.4
No reason	129	76.8
Others	19	11.3
Awareness of cancer screening center	327	84.3

Multiple answers allowed.

**Table 5:** Association between sociodemographic variables and utilization of cervical cancer screening services.

Variables	Total number	Have been screened		Chi-square	Df	P value
		Yes	No			
Age						
≤40	342	80	262	13.56	1	0.001*∗*
>40	46	0	46			
Marital status						
Married	228	80	148	70.72	1	0.001*∗*
Unmarried	160	0	160			
Religion						
Christianity	384	80	304	1.86	1	0.424*∗∗*
Islam	4	0	4			
Parity						
≤4	369	80	289	9.03	1	0.013*∗∗*
]=>4	19	0	19			
Duration of practice						
<5	183	80	103	111.90	1	0.001*∗*
≥5	205	0	205			

*∗*Pearson Chi-square. *∗∗*Likelihood ratio.

## Data Availability

The data used to support the findings of this study are included within the article.
